# Characterisation of runs of homozygosity and inbreeding coefficients in the red-brown Korean native chickens

**DOI:** 10.5713/ab.23.0514

**Published:** 2024-04-25

**Authors:** John Kariuki Macharia, Jaewon Kim, Minjun Kim, Eunjin Cho, Jean Pierre Munyaneza, Jun Heon Lee

**Affiliations:** 1 Division of Animal and Dairy Science, Chungnam National University, Daejeon 34134, Korea; 2 Department of Bio-AI Convergence, Chungnam National University, Daejeon 34134, Korea

**Keywords:** Inbreeding Coefficient, ROH Island, Run of Homozygosity, Selection Signatures

## Abstract

**Objective:**

The analysis of runs of homozygosity (ROH) has been applied to assess the level of inbreeding and identify selection signatures in various livestock species. The objectives of this study were to characterize the ROH pattern, estimate the rate of inbreeding, and identify signatures of selection in the red-brown Korean native chickens.

**Methods:**

The Illumina 60K single nucleotide polymorphism chip data of 651 chickens was used in the analysis. Runs of homozygosity were analysed using the PLINK v1.9 software. Inbreeding coefficients were estimated using the GCTA software and their correlations were examined. Genomic regions with high levels of ROH were explored to identify selection signatures.

**Results:**

A total of 32,176 ROH segments were detected in this study. The majority of the ROH segments were shorter than 4 Mb. The average ROH inbreeding coefficients (*F_ROH_*) varied with the length of ROH segments. The means of inbreeding coefficients calculated from different methods were also variable. The correlations between different inbreeding coefficients were positive and highly variable (*r* = 0.18–1). Five ROH islands harbouring important quantitative trait loci were identified.

**Conclusion:**

This study assessed the level of inbreeding and patterns of homozygosity in Red-brown native Korean chickens. The results of this study suggest that the level of recent inbreeding is low which indicates substantial progress in the conservation of red-brown Korean native chickens. Additionally, Candidate genomic regions associated with important production traits were detected in homozygous regions.

## INTRODUCTION

Indigenous chickens are important genetic resources due to their adaptive capacity and disease resilience. Thus, their conservation is essential for the improvement of poultry breeds. However, the existence of these indigenous breeds is threatened by the rapid introduction of high-producing commercial chicken breeds [1]. The Korean native chickens have inhabited the Korean Peninsula for many years, becoming a remarkable genetic resource in the region [2]. The chickens underwent a severe bottleneck during the Korean War, threatening their existence. Since then, restoration and conservation programs have been implemented [3].

The red-brown chicken breed is among the five lines of Korean native chickens which are distinguishable by their feather colour [4]. Despite their slow growth rate, Korean native chickens have recently gained popularity due to their superior meat quality traits. Thus, they are potential resources in meeting the expanding demand for poultry products [2]. The red-brown line is reported to have favourable carcass quality traits and has been crossbred with exotic broiler chickens to improve meat production [4,5].

Genetic conservation programs should preserve alleles and allelic combinations and maximise heterozygosity in populations [4,6]. The progress in genetic conservation is assessed through the estimation of genetic diversity parameters [7]. The inbreeding coefficient is widely used in the estimation of genetic variability in livestock populations [8]. It is defined as the probability that two alleles at a locus are inherited from a common ancestor [9].

Conventionally, inbreeding coefficients in livestock populations have been calculated using pedigree data. However, these estimates are often unreliable due to the scarcity of pedigree information and inaccurate records [10]. The impreciseness of pedigree-based inbreeding estimation and the availability of large amounts of genomic data have inspired the application of genomic-based estimation of inbreeding coefficients [10,11]. Genomic inbreeding coefficients are more reliable than pedigree-based coefficients because they capture ancient inbreeding and account for the random inheritance of alleles [11]. Additionally, the level of genetic variability in specific genomic regions can be revealed.

Several approaches have been applied in the estimation of genomic inbreeding coefficients. These include methods based on runs of homozygosity (ROH), excess homozygosity, diagonal elements of genomic relationship matrices and correlation between alleles in uniting gametes [10]. ROH-based inbreeding coefficients are widely used because they tend to have a strong correlation with pedigree-based inbreeding coefficients and reflect the homozygosity level due to inbreeding [12].

ROHs are long homozygous DNA segments that form due to the inheritance of identical haplotypes from the same ancestor [13]. Their characterization is important in revealing the breeding history of a population. The analysis of ROH has been conducted on various livestock species, including chickens [14–18]. Indeed, the impact of inbreeding on important livestock traits has been demonstrated through studies on ROH. For instance, the negative effects of inbreeding on production and fertility traits in dairy cattle have been investigated through studies on ROH [15–18].

ROH analyses have also been employed to explore the impact of selection on the genetic diversity of animals. In chickens, investigations of the ROH have been conducted to assess the patterns of inbreeding and identify selection signatures in various native and commercial lines [16,17]. Using high-density single nucleotide polymorphisms (SNPs) markers, the patterns of ROH in Chinese indigenous chickens were characterized and candidate genes and quantitative trait locus (QTL) related to body weight, feed intake and muscle development on the ROH islands were identified [19].

The genetic diversity of Korean indigenous chickens has been examined using microsatellite and SNP markers [7,20]. Previous studies have mainly focused on the characterization of population structure, linkage disequilibrium decay and phylogenetics. These parameters reflect the Euclidean distance between individuals in a population [21]. Estimation of relatedness and inbreeding coefficients in Korean native chickens using genomic data has not been comprehensively conducted. In this regard, this study aimed to characterise the ROH, estimate inbreeding coefficients, and identify candidate genes and QTLs in homozygous genomic regions in the red-brown Korean native chicken.

## MATERIALS AND METHODS

### Ethical statement

The care and handling of animals used in this study were approved by the Institute of Animal Care and Use Committee of the National Institute of Animal Science (approval No: NIAS 20212219) and Chungnam National University’s Animal Ethics Committee.

### Study samples, genotyping, and quality control

The study animals were obtained from the red-brown Korean native chicken pure line reared at the National Institute of Animal Science, Republic of Korea. The study sample consisted of a total of 651 chickens sampled from four consecutive generations between the years 2019 and 2022. The number of samples per generation is shown in Table 1. Genomic DNA was then extracted from the blood samples using a commercial kit (GeNet Bio, Daejeon, Korea). The DNA samples were genotyped using the Illumina chicken 60K SNP chip (Illumina Inc., San Diego, CA, USA) containing 57,636 SNPs [22]. Sex chromosomes and markers that were not assigned to specific chromosomes were excluded from further analyses. Only SNPs with a call rate (>0.9) and Handy-Weinberg equilibrium (p-value >1.0×10^−6^) were used for the analysis. No further quality control was conducted during the analysis of ROH, as recommended by Meyermans et al [23]. On the other hand, genotypes with a minor allele frequency of less than 0.05 were excluded during the estimation of other inbreeding coefficients. Consequently, 53,872 SNPs were used for ROH analysis while 44,569 SNPs were available for the calculation of other inbreeding coefficients.

### Analysis of runs of homozygosity

The calling of ROH was conducted using the (--*homozyg*) function in PLINK v1.9 software [24]. The analysis parameters were set to ensure maximum genome coverage and minimise calling errors. Consequently, ROH segments were called based on the following parameters: a minimum ROH length of 1,000 Kb, a sliding window with a minimum of 49 SNPs, one heterozygous SNP per ROH segment, five missing SNPs per ROH segment, a maximum gap of 1,500 Kb between consecutive homozygous SNPs and the threshold of overlapping homozygous windows was set to 0.05. The minimum number of SNPs in the sliding window was estimated based on the formula proposed by Lencz et al [25] as follows:


$$L=\frac{{\text{log}}_{e}\;\alpha /n_{s}n_{i}}{{\text{log}}_{e}(1-het)}$$

Where, *n_s_* is the number of genotyped SNPs per sample, *n_i_* is the number of genotyped individuals, α is the percentage of false positive ROH (0.05) and *het* is the average heterozygosity across all genotyped SNPs. The ROH segments were grouped into classes based on length. The ROH categories were: 1 to 2, 2 to 4, 4 to 8, 8 to 16 and >16 Mb. The patterns of ROH were reported in terms of the mean ROH length per individual, the minimum, average and maximum length of ROH segments, and the frequency of ROH segments in different ROH length classes. The ROH summary statistics were calculated using the detectRUNS package in R software [26]. The ROH pattern across the four generations was also scrutinized.

### Estimation of inbreeding coefficients

The coefficient of inbreeding based on ROH was calculated using a formula employed by McQuillan et al [27] as follows:


$$F_{ROH}=\frac{\Sigma \;L_{ROH}}{L_{AUTO}}$$

Where, *L_ROH_* is the sum of the length of ROH segments in the genome of each bird and *L_AUTO_* is the total length of the autosomal genome. The genome length was estimated to be 0.94 Gb. The *F_ROH_* was also calculated for the different ROH classes defined based on the ROH length.

Additionally, three other measurements of inbreeding were estimated using the (*--ibc*) function in the GCTA software [28]. These include:

Inbreeding coefficient calculated from the diagonals of the genomic kinship matrix (*F_GRM_*) [29] as follows:


$$F_{GRM}=\frac{ZZ^{\prime }}{2\;\Sigma \;p_{j}(1-p_{j})}-1$$

Where, Z is a covariance matrix constructed by subtracting twice the minor allele frequency from the raw marker score. The covariance matrix consists of values: (0-2p) for homozygous, (1-2p) for heterozygous and (2-2p) for alternate homozygous loci, where p is the minor allele frequency.

The Wright’s inbreeding coefficient (*F_HOM_*) which compares the expected and the observed homozygous genotypes for each sample [28] as follows:


$$F_{HOM}=\frac{Number\;of\;observed\;homozygous\;loci-number\;of\;expected\;homozygous\;loci}{Number\;of\;nonmissing\;loci-number\;of\;expected\;homozygous\;loci}$$

Wright’s inbreeding coefficients calculated as the correlation between alleles in uniting gametes (*F_UNI_*) [28]:


$$F_{UNI}=\frac{1}{n}\sum_{m=1}^{n}\frac{x_{m-(1+2p_{m})\;x_{m}+2p_{m}^{2}}^{2}}{2p_{m}q_{m}}$$

Where, *x* is the number of versions of the reference allele, p and q are the reference and alternative allele frequencies of *m*th SNP respectively. The correlation between *F_ROH_* coefficients and other inbreeding coefficients was estimated using Pearson’s correlation and plotted using the Corrplot package in R software [26]. The trend in average inbreeding coefficients across the four generations was also evaluated.

### Identification and functional annotation of genes and quantitative trait locus in runs of homozygosity islands

Genomic regions with common ROH across the population were extracted using the detectRUNS package in R software. The frequency of occurrence of SNPs in the ROH segments was estimated by enumerating the number of times each SNP was detected in the ROH. Genomic regions where the incidence of SNP in ROH exceeded a population-specific threshold were considered ROH islands. The threshold was calculated as described by Gorssen et al [30]. Accordingly, the distribution of ROH incidences was standardized using a standard normal Z-score and then the top 0.1% of SNPs with a p>0.999 were selected to form the ROH islands. The GALLO package in R software was used to identify annotated genes and QTLs in the ROH islands [31]. The QTLs in the ROH islands were annotated based on the animal QTL annotation database [32]. Similarly, candidate genes in the ROH islands were annotated based on the GRCg6a reference genome assembly. Finally, a literature search was conducted to elucidate the functions of the identified genes.

## RESULTS

### Runs of homozygosity

**Figure 1:**
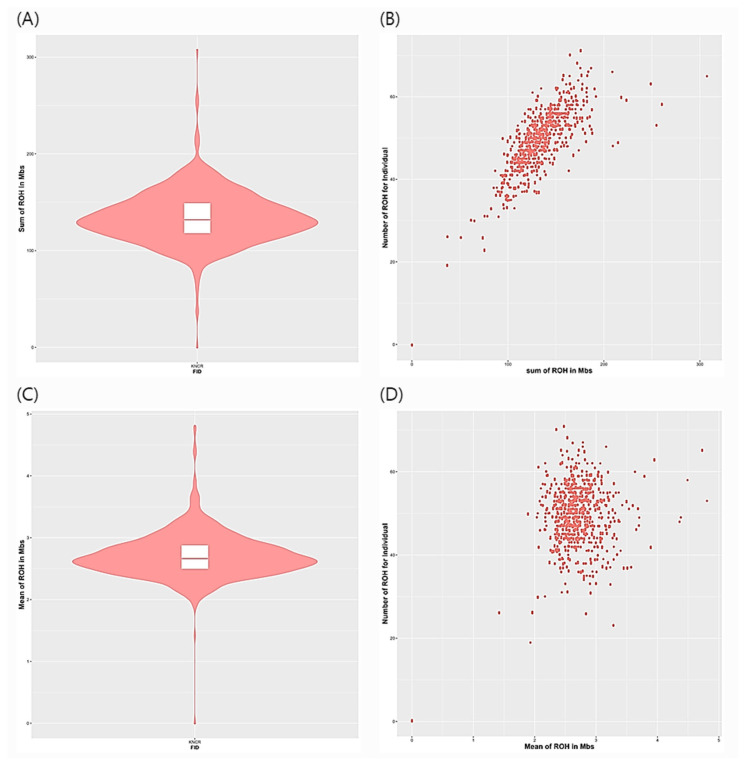
Comparison of the length and number of ROH segments in red-brown Korean native chickens. (A) Total sum of ROH for individuals; (B)Scatterplot of number of ROH in individuals versus sum ROH length; (C) Average length of ROH segments for individuals; (D) Scatterplot of the number of ROH for individuals versus the average length of ROH segment. ROH, runs of homozygosity.

**Figure 2:**
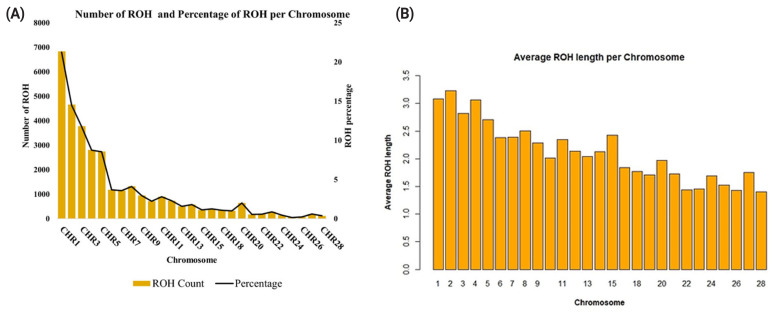
Characterization of ROH in chromosomes. (A) The bars represent the number of ROH while the line represents the percentage of ROH per chromosome; (B) Average length of ROH per chromosome. ROH, runs of homozygosity.

Genotype data was utilised to characterise the ROH and estimate inbreeding coefficients in the red-brown Korean native chicken. Figure 1 shows the distribution of the length and numbers of ROH segments. A total of 32,176 ROH segments were called, with an average of 49 segments per bird. The average length of the ROH segments was 2.7 Mb. The longest ROH segment was found on *Gallus gallus* chromosome (GGA) 2 with a length of 33.47 Mb, while the shortest segment was identified on GGA1 with a length of 1.00 Mb. GGA1 had the highest number of ROH segments (n = 6,832) while GGA2 had the greatest average ROH length (3.2 Mb) (Figures 2A–2B). The overall average ROH length was 134.12 Mb (Figure 1A).

**Figure 3:**
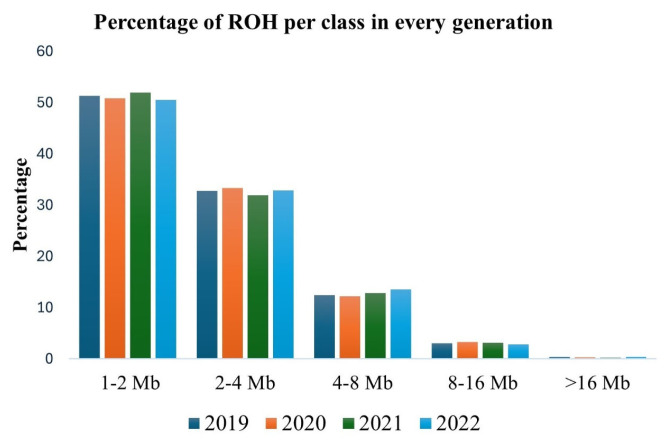
Percentage of ROH segments in each ROH category per generation. ROH, runs of homozygosity.

The ROH segments were further classified based on their length into 1 to 2, 2 to 4, 4 to 8, 8 to 16, and >16 Mb classes (Table 2). Short ROH segments (1 to 4 Mb) were predominant (83.84%). In contrast, long ROH segments (>8 Mb) were few (3.36%). ROH segments of moderate length (4 to 8 Mb) accounted for only 12.75% of all ROH found. There were no marked differences in the percentage of ROH segments in each ROH category across the four generations (Figure 3).

### Genomic inbreeding coefficients

**Figure 4:**
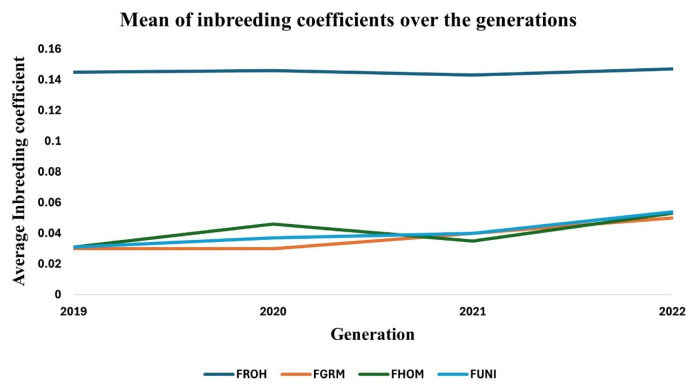
Trend of average inbreeding coefficients across the generations. FROH, FGRM, and FUNI are inbreeding coefficients calculated using methods based on ROH, genomic relation matrix, excess homozygosity, and correlation between uniting gametes, respectively.

The inbreeding coefficients estimated from the different methods and classes of ROH are summarised in Table 3. The overall average inbreeding coefficient (*F_ROH_*) ranged between 0.039 and 0.327. The other inbreeding coefficients varied between [−0.232 to 3.591], [−0.441 to 0.951], and [−0.337 to 2.271] for *F_GRM_*, *F_HOM_*, and *F_UNI_* respectively. The *F_ROH_* was also calculated for the different ROH length classes. The average *F_ROH_* (1 to 2 Mb) was equal to the average *F_ROH_* values. The *F_ROH_* coefficients estimated from segments longer than 4 Mb were smaller than the overall *F_ROH_*. Figure 4 shows the trends of average inbreeding coefficients across the four generations. There was no marked difference in average *F_ROH_* across the four generations.

**Figure 5:**
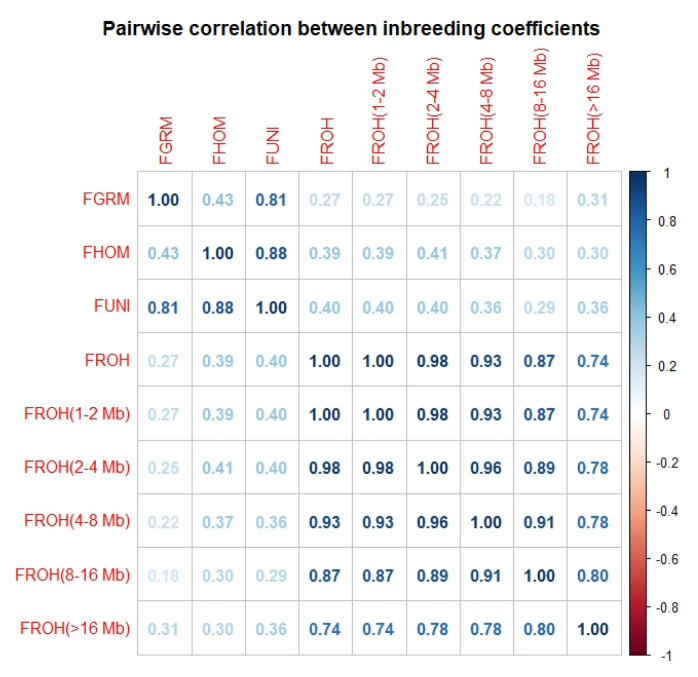
Pairwise correlations between estimated inbreeding coefficients in red-brown Korean native chicken population. These included inbreeding coefficients calculated from the diagonal elements of the genomic relationship matrix (FGRM), excess homozygosity (FHOM), correlation of alleles between uniting gametes (FUNI), and runs of homozygosity (FROH).

The correlations between the inbreeding coefficients were all positive but highly variable (Figure 5). All the *F_ROH_* estimates were strongly correlated (r>0.74) while correlations between the *F_ROH_* and other inbreeding coefficients were low to moderate. The highest correlation was found between *F_ROH_* and *F_UNI_* (*r* = 0.41) while *F_ROH_* (4 to 8 Mb) and *F_GRM_* had the lowest correlation (*r* = 0.18). There was a strong correlation between *F_GRM_* and *F_UNI_* (*r* = 0.81) and between *F_HOM_* and *F_UNI_* (*r* = 0.88). The correlation between *F_GRM_* and *F_HOM_* was relatively weak (*r* = 0.41).

### Runs of homozygosity islands and identification of selection signatures

**Figure 6:**
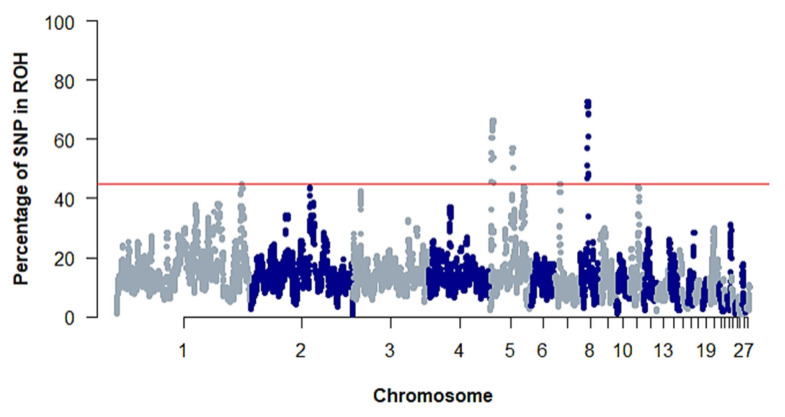
Manhattan plot for the occurrence of SNPs in ROH islands in the red-brown Korean native chicken population. The red line shows the threshold for selecting the top ROH islands (44.7%). SNPs, single nucleotide polymorphisms; ROH, runs of homozygosity.

**Figure 7:**
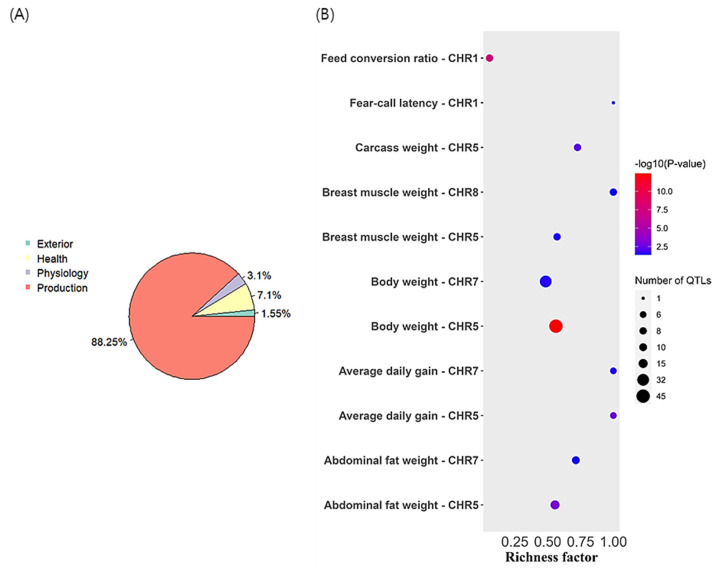
The summary of QTLs in ROH islands in the red-brown Korean native chicken population (A) Proportion of annotated QTLs for different traits, (B) Bubble plot depicting the top enriched QTLs (adjusted p-value<0.05). QTLs, quantitative trait locus.

Common homozygous genomic regions in the study population (ROH islands) were explored by selecting the top 0.1% of the SNPs found in the ROH segments in 44.7% of the population. Consequently, five ROH islands in GGA1, GGA5, GGA7, and GGA8 were identified (Figure 6). The longest ROH was on GGA8 with a length of 2.3 Mb and a total of 114 SNPs, while the shortest ROH was on GGA1 with a length of 0.05 Mb and only four SNPs. The ROH islands had 88 annotated protein-coding genes (Table 4). Functional enrichment of the identified genes did not reveal any significant gene ontology terms probably due to the small number of genes in the ROH islands. Consequently, a literature review was conducted to elucidate the functions of the genes identified. Notable genes identified in the ROH islands included *NELL1*, *BDNF, COL6A1*, *AMYA1*, *PLAG4S*, and *PTGS2*. The ROH islands were mapped to the chicken QTL database to identify overlapping QTLs, resulting in 11 enriched QTLs (adjusted p-value<0.05). Of the identified QTLs, 88.25% were associated with important production traits such as body weight, abdominal fat weight, carcass weight and feed conversion ratio (Figure 7A). The top enriched QTLs found per chromosome are depicted in Figure 7B. The ROH island in GGA1 overlapped with QTLs related to body weight and daily weight gain in chickens. GGA5 harbored the highest number of QTLs in the ROH islands which included the QTLs linked to body weight, carcass weight and abdominal fat content. Similarly, the ROH islands in GGA7 and GGA8 contained QTLs related to carcass weight and feed intake.

## DISCUSSION

### Runs of homozygosity

The success of genetic conservation programs relies on the maintenance of genetic variability in a population. The rising demand for livestock products has led to increased selection intensity for higher production, resulting in inbreeding [33]. This can lead to low genetic variability and consequently reduce the population fitness and slow genetic gain in production traits [34]. In the current study, the patterns of ROH in the red-brown Korean native chickens were analysed to assess the extent of inbreeding and genetic variability. Additionally, the ROH islands were evaluated for candidate homozygous regions having genes and QTLs of interest in chicken production.

The length and distribution of ROH segments can give insights into the population’s genetics and breeding history [13]. Homozygous genomic segments inherited from the same ancestor are fragmented into smaller segments by recombination over generations [35]. Thus, short ROH segments indicate ancient inbreeding, while longer ROH segments are due to recent inbreeding.

In this study, short ROH segments less than 4 Mb were prevalent across the four generations, indicating that Korean native chickens may have experienced a bottleneck and inbreeding in the past. The short ROH segments may be due to the impact of other events such as recombination, population contraction and genetic drift. However, it is important to note that the use of medium-density SNP data tends to exaggerate the number of short ROH segments (<4 Mb) [36]. Therefore, the number of short ROH segments in this study might be overestimated. Generally, previous studies including those using whole genome sequences, have reported higher proportions of short ROH segments [19,37]. This suggests that short ROH segments are common in the chicken genome probably because of recombination. On the other hand, the low proportion of long ROH (>8 Mb) indicates that recent inbreeding is uncommon in the population which may suggest good progress in the conservation of genetic diversity. The ROH distribution pattern per chromosome revealed that the number and size of the ROH segments varied with chromosomal size. As expected, the macrochromosomes (GGA1–GGA5) had more and longer ROH segments compared to the smaller chromosomes.

### Genomic inbreeding coefficients

Conventionally, the level of inbreeding is estimated using pedigree information [34]. However, inaccurate pedigree records and missing information can limit the accuracy of the estimated inbreeding coefficients. Furthermore, estimates from pedigree records do not account for Mendelian sampling effects. The recent advancement in genomic sequencing technology has enabled the generation of large amounts of genomic data. Thus, genomic inbreeding coefficients can be reliably estimated. Genomic inbreeding coefficients do not depend on pedigree records hence not affected by recording errors. Additionally, these estimates give account to Mendelian sampling effects, making them more realistic [12].

Four types of inbreeding coefficients were calculated and compared in this study. There was no significant change in average *F_ROH_* across the four generations. This further shows that the rate of recent inbreeding in the red-brown Korean chickens is low. The estimated inbreeding coefficients were considerably variable. Unlike other coefficients, the *F_ROH_* estimates ranged between 0 and 1 which is in line with the true definition of the inbreeding coefficient [9]. The average *F_ROH_* calculated from ROH segments shorter than 4 Mb was higher than *F_ROH_* from longer segments. This could be due to the overestimation of short ROH segments [36]. The *F_GRM_*, *F_HOM_*, and *F_UNI_* estimates ranged between negative values and values greater than one. Negative inbreeding coefficients suggest a gain in genetic variability [10]. On the other hand, Inbreeding coefficients greater than one are difficult to interpret because it is not practical to lose more than 100% of the genetic variation [14]. Unlike *F_ROH_*, the three SNP by SNP inbreeding coefficients are dependent on allele frequencies in the population. They only yield reasonable estimates when the allele frequencies in the population are equal to those of the base population [12]. Additionally, these methods do not differentiate genomic regions that are identical by descent from those identical by state [12]. *F_ROH_* could be regarded as a more reliable measure of inbreeding since it is robust to allele frequencies and only reflects the homozygosity that is identical by descent [12].

All the inbreeding coefficients were positively correlated. The overall *F_ROH_* value was strongly correlated with *F_ROH_* (1 to 2 Mb) and *F_ROH_* (2 to 4 Mb) (*r*>0.98). This suggests that a large proportion of inbreeding in the study population is ancient. This is consistent with previous studies in Chinese indigenous chickens [19]. The correlation between *F_ROH_* and other coefficients was low to moderate. The correlation between *F_ROH_* and *F_UNI_* was higher compared to other inbreeding coefficients (*r* = 0.41). The low correlation between *F_ROH_* and other inbreeding coefficients could be due to discrepancies in allele frequencies and the different approaches used in the calculation [12,37]. *F_GRM_* was highly correlated with *F_HOM_*. Likewise, the correlation between *F_GRM_* and *F_UNI_* was strong. This is consistent with previous reports on chicken and cattle [16,37].

### Runs of homozygosity islands and identification of selection signatures

Selection pressure increases the frequency of homozygous genotypes in a population, thus increasing the homozygosity rate at a given locus and neighbouring sites [38]. Common ROH segments in a population can therefore reveal how selection has modified the variability of specific genomic regions. The current study identified five ROH islands that were common in 44.7% of the study population. Functional annotation of these regions found QTLs associated with production traits, including, body and carcass weight, breast muscle weight, abdominal fat weight and daily weight gain. Tian et al [19] mapped similar QTLs in the ROH islands of indigenous Chinese chickens. These results indicate the loss of genetic variability in genomic regions associated with production traits in chickens probably due to selection.

In this study, annotated genes coding for proteins and long non-coding RNAs were identified in the ROH islands. The ROH islands in GGA5 harboured notable genes including the neural EGFL like 1 (*NELL1*) and *BBOX1*, which are linked to the regulation of bone development and feed efficiency in chickens respectively [39–41]. Another important gene located on the ROH island of GGA5 was the leucine-rich repeat-containing G protein-coupled receptor (*LGR4*) which is involved in the regulation of carbohydrates, lipids and amino acid metabolic pathways [42]. In the current study, the *LGR4* gene was mapped in the ROH island overlapping with QTLs associated with growth performance traits. This suggests that the gene may be important in the growth performance of chickens. The same ROH island harboured the brain-derived neurotrophic factor (*BDNF*) gene which is associated with responses to environmental stressors such as heat stress and light intensity in chickens [43].

The collagen type 11 alpha 1 chain (*COL6A1*) gene located in ROH island in GGA7 is highly expressed in the growing Graafian follicles of laying chickens [44]. This gene is reported to influence intramuscular fat deposition in chickens; thus, it may affect meat quality [45]. The ROH island in GGA8 contained notable genes, such as amylase alpha 1 (*AMYA1*) which encodes for the amylase enzyme that is important in starch metabolism [46]. Notably, the same ROH island overlapped with QTL associated with breast muscle weight which suggests that the *AMYA1* gene could be important in the growth performance of chickens. This gene was also mapped in the ROH island of Chinese indigenous chickens [19]. Additionally, the ROH island in GGA8 contained the *PLA2G4A* and *PTGS2* genes, which have been linked to reproductive functions in various livestock species [47]. Interestingly, some of the identified genes have been reported as selection signatures in other chicken breeds [16,19]. This suggests that there are common genomic regions that are subject to natural and artificial selection across various breeds of chicken.

## CONCLUSION

This study revealed the distribution pattern of ROH in the red-brown Korean native chickens. The low prevalence of long ROH segments suggests minimal recent inbreeding. Moreover, there was no marked change in means of inbreeding coefficients across the four generations analysed. This shows substantial progress in the conservation of red-brown Korean chickens. Additionally, candidate genomic regions associated with important production traits were detected in the ROH islands. In summary, this study provides insights into the inbreeding history and genetic characteristics of the red-brown Korean native chickens.

## Figures and Tables

**Table 1:** Number of samples per generation

Generation	Sample size
2019	158
2020	193
2021	100
2022	200

**Table 2:** Summary of runs of homozygosity segments categorized into different classes based on length

ROH class (Mb)	ROH count	ROH frequency	Mean ROH (Mb)
1–2	16,414	0.511	1.441
2–4	10, 566	0.327	2.732
4–8	4,110	0.128	5.504
8–16	981	0.032	10.267
>16	105	0.003	20.085

ROH, runs of homozygosity.

**Table 3:** Summary of inbreeding coefficients calculated using different methods and runs of homozygosity classes

Inbreeding coefficient	Minimum	Average	Maximum
*F* *_GRM_*	−0.232	0.045	3.591
*F* *_HOM_*	−0.441	0.044	0.951
*F* *_UNI_*	−0.337	0.044	2.271
Overall, *F_ROH_*	0.039	0.143	0.327
*F_ROH_* (1–2 Mb)	0.039	0.143	0.327
*F_ROH_* (2–4 Mb)	0.002	0.104	0.291
*F_ROH_* (4–8 Mb)	0.006	0.057	0.220
*F_ROH_* (8–16 Mb)	0.009	0.025	0.180
*F_ROH_* (>16 Mb)	0.017	0.026	0.094

ROH, runs of homozygosity; *F_GRM_*, genomic relationship matrix; *F_HOM_*, excess homozygosity; *F_UNI_*, the correlation of alleles between uniting gametes; *F_ROH_*, inbreeding coefficient from ROH.

**Table 4:** Identified genes in the runs of homozygosity islands of red-brown Korean native chickens

Chromosome	N of SNPs	Start position	End position	Genes
1	4	2,094,372	183977056	*YAP1, BIRC3*
5	91	2,094,372	4,419,350	*NAV2, LEUXT, PRMT3, ENSGALG00000047154, SLC6A5, NELL11, ENSGALG00000051731, ENSGALG00000052381, ANOS5, SLC17A6, FANCF, GAS2, SVIP, ANOS3, SLC5A12, FIBIN, BBOX1, LGR4, LIN7C, BDNF, KIF18A, METTL15P1, ENSGALG00000048701*
5	55	32,275,187	33,710,945	*DPH6, ZNF770, AQR, ACTC1, GJD2, STXBP6, NOVA1, ENSGALG00000053993*
7	52	6,767,284	8,224,314	*ENSGAL000000050048, PCNT, C210rf58, KMO, ITGB2, ENSGAL00000007513, ADARB1, GLS2, STAT1, STAT4, MYO1B, ENSGAL000000052218, NABP1, CAVIN2, TMEFF2*
8	114	10,359,540	12,687,857	*ENSGALG00000047029, PLA2G4A, PTGS2, PDC, C8H1orf27, TPR, ENSGALG00000053885, HMCN1, IVNS1ABP, SWT1, TRMT1L, AMY1A, RNPC3, ENSGALG00000046817, ENSGALG00000005180, ENSGALG00000052635, OLFM3, ENSGALG00000047706, S1PR1, ENSGALG00000020884, ENSGALG00000025580, DPH5, SLC30A7, EXTL2, CDC14A, GPR88, ENSGALG00000005257, RTCA, DBT, LRRC39, TRMT13, SASS6, MFSD14A, SLC35A3, ENSGALG00000046652, AGL, FRRS1, PALMD, PLRPPR5, PLPPR4*

SNPs, single nucleotide polymorphisms.

## Data Availability

The datasets used in this study can be availed upon request to the corresponding author.

## References

[1] Cendron F, Mastrangelo S, Tolone M, Perini F, Lasagna E, Cassandro M (2021). Genome-wide analysis reveals the patterns of genetic diversity and population structure of 8 Italian local chicken breeds. Poult Sci.

[2] Cha J, Choo H, Srikanth K (2021). Genome-wide association study identifies 12 loci associated with body weight at age 8 weeks in Korean native chickens. Genes.

[3] Jin S, Park HB, Seo DW (2014). Association of MC1R genotypes with shank color traits in Korean native chicken. Livest Sci.

[4] Jung S, Bae YS, Kim HJ (2013). Carnosine, anserine, creatine, and inosine 5′-monophosphate contents in breast and thigh meats from 5 lines of Korean native chicken. Poult Sci.

[5] Kim M, Munyaneza JP, Cho E (2023). Genome-wide association study on the content of nucleotide-related compounds in Korean native chicken breast meat. Animals (Basel).

[6] Caballero A, Rodríguez-Ramilo ST, Ávila V, Fernández J (2009). Management of genetic diversity of subdivided populations in conservation programmes. Conserv Genet.

[7] Suh S, Sharma A, Lee S (2014). Genetic diversity, and relationships of Korean chicken breeds based on 30 microsatellite markers. Asian-Australas J Anim Sci.

[8] Krupa E, Žáková E, Krupová Z (2015). Evaluation of inbreeding and genetic variability of five pig breeds in czech republic. Asian-Australas J Anim Sci.

[9] Wright S (1922). Coefficients of inbreeding and relationship. Am Nat.

[10] Villanueva B, Fernández A, Saura M (2021). The value of genomic relationship matrices to estimate levels of inbreeding. Genet Sel Evol.

[11] Keller MC, Visscher PM, Goddard ME (2011). Quantification of inbreeding due to distant ancestors and its detection using dense single nucleotide polymorphism data. Genetics.

[12] Zhang Q, Calus MP, Guldbrandtsen B, Lund MS, Sahana G (2015). Estimation of inbreeding using pedigree, 50k SNP chip genotypes and full sequence data in three cattle breeds. BMC Genetics.

[13] Ceballos FC, Joshi PK, Clark DW, Ramsay M, Wilson JF (2018). Runs of homozygosity: windows into population history and trait architecture. Nat Rev Genet.

[14] Dadousis C, Ablondi M, Cipolat-Gotet C (2022). Genomic inbreeding coefficients using imputed genotypes: assessing different estimators in Holstein-Friesian dairy cows. J Dairy Sci.

[15] Bjelland DW, Weigel KA, Vukasinovic N, Nkrumah JD (2013). Evaluation of inbreeding depression in Holstein cattle using whole-genome SNP markers and alternative measures of genomic inbreeding. J Dairy Sci.

[16] Huang X, Otecko NO, Peng M (2020). Genome-wide genetic structure, and selection signatures for color in 10 traditional Chinese, yellow-feathered chicken breeds. BMC Genomics.

[17] Fedorova ES, Dementieva NV, Shcherbakov YS, Stanishevskaya OI (2022). Identification of key candidate genes in runs of homozygosity of the genome of two chicken breeds, associated with cold adaptation. Biology.

[18] Ferenčaković M, Hamzić E, Gredler B (2012). Estimates of autozygosity derived from runs of homozygosity: empirical evidence from selected cattle populations. J Anim Breed Genet.

[19] Tian S, Tang W, Zhong Z (2023). Identification of runs of homozygosity islands and functional variants in Wenchang Chicken. Animals (Basel).

[20] Seo D, Lee DH, Choi N, Sudrajad P, Lee SH, Lee JH (2018). Estimation of linkage disequilibrium and analysis of genetic diversity in Korean chicken Lines. PLoS ONE.

[21] Salojärvi J, Rajora OP (2018). Computational tools for population genomics. Population Genomics.

[22] Groenen MA, Megens HJ, Zare Y (2011). The development and characterization of a 60k SNP chip for chicken. BMC Genomics.

[23] Meyermans R, Gorssen W, Buys N, Janssens S (2020). How to study runs of homozygosity using PLINK? A guide for analyzing medium density SNP data in livestock and pet species. BMC Genomics.

[24] Chang CC, Chow CC, Tellier LC, Vattikuti S, Purcell SM, Lee JJ (2015). Second-generation PLINK: Rising to the challenge of larger and richer datasets. GigaScience.

[25] Lencz T, Lambert C, DeRosse P (2007). Runs of homozygosity reveal highly penetrant recessive loci in schizophrenia. Proc Natl Acad Sci USA.

[26] (c2023). The R project for statistical computing [Internet].

[27] McQuillan R, Leutenegger AL, Abdel-Rahman R (2008). Runs of homozygosity in European populations. Am J Hum Genet.

[28] Yang J, Lee SH, Goddard ME, Visscher PM (2011). GCTA: a tool for genome-wide complex trait analysis. Am J Hum Genet.

[29] VanRaden PM, Olson KM, Wiggans GR, Cole JB, Tooker ME (2011). Genomic inbreeding and relationships among Holsteins, jerseys, and Brown Swiss. J Dairy Sci.

[30] Gorssen W, Meyermans R, Janssens S, Buys N (2021). A publicly available repository of Roh Islands reveals signatures of selection in different livestock and pet species. Genet Sel Evol.

[31] Fonseca PAS, Suárez-Vega A, Marras G, Cánovas Á (2020). Gallo: an R package for genomic annotation and integration of multiple data sources in livestock for positional candidate loci. GigaScience.

[32] Hu Z (c2024). Animal QTL database [Internet].

[33] Wu X, Zhou R, Zhang W (2021). Genome-wide scan for runs of homozygosity identifies candidate genes in Wannan Black pigs. Anim Biosci.

[34] Xue Q, Li G, Cao Y (2021). Identification of genes involved in inbreeding depression of reproduction in Langshan chickens. Anim Biosci.

[35] Wang Q, Zhang J, Wang H (2023). Estimates of genomic inbreeding and identification of candidate regions in Beijing-You chicken populations. Anim Genet.

[36] Ferenčaković M, Sölkner J, Curik I (2013). Estimating autozygosity from high-throughput information: effects of SNP density and genotyping errors. Genet Sel Evol.

[37] Ferenčaković M, Hamzić E, Gredler B (2012). Estimates of autozygosity derived from runs of homozygosity: empirical evidence from selected cattle populations. J Anim Breed Genet.

[38] Hewett AM, Stoffel MA, Peters L, Johnston SE, Pemberton JM (2023). Selection, recombination, and population history effects on runs of homozygosity (ROH) in wild red deer (cervus elaphus). Heredity.

[39] Xue J, Peng J, Yuan M (2011). NELL1 promotes high-quality bone regeneration in rat femoral distraction osteogenesis model. Bone.

[40] Lee J, Karnuah AB, Rekaya R, Anthony NB, Aggrey SE (2015). Transcriptomic analysis to elucidate the molecular mechanisms that underlie feed efficiency in meat-type chickens. Mol Genet Genom.

[41] Gao C, Du W, Tian K (2023). Analysis of conservation priorities and runs of homozygosity patterns for Chinese indigenous chicken breeds. Animals (Basel).

[42] Li Z, Zhang W, Mulholland MW (2015). LGR4 and its role in intestinal protection and energy metabolism. Front Endocrinol.

[43] Yang Y, Cong W, Liu J (2022). Constant light in early life induces fear-related behavior in chickens with suppressed melatonin secretion and disrupted hippocampal expression of clock- and BDNF-associated genes. J Anim Sci Biotechnol.

[44] Zhou S, Ma Y, Zhao D, Mi Y, Zhang C (2020). Transcriptome profiling analysis of underlying regulation of growing follicle development in the chicken. Poult Sci.

[45] Zhang M, Li D, Zhai Y (2020). The landscape of DNA methylation associated with the transcriptomic network of intramuscular adipocytes generates insight into intramuscular fat deposition in chicken. Front Cell Dev Biol.

[46] Zhang Z, Zhong H, Lin S (2021). Polymorphisms of amy1a gene and their association with growth, carcass traits and feed intake efficiency in chickens. Genomics.

[47] Bernini F, Bagnato A, Marelli SP, Zaniboni L, Cerolini S, Strillacci MG (2021). Genetic diversity, and identification of homozygosity-rich genomic regions in seven Italian heritage turkey (Meleagris gallopavo) breeds. Genes.

